# A Novel Asynchronous Brain Signals-Based Driver–Vehicle Interface for Brain-Controlled Vehicles

**DOI:** 10.3390/bioengineering10091105

**Published:** 2023-09-21

**Authors:** Jinling Lian, Yanli Guo, Xin Qiao, Changyong Wang, Luzheng Bi

**Affiliations:** 1 Beijing Institute of Basic Medical Sciences, 27 Taiping Rd., Beijing 100850, China; lianjinling@bit.edu.cn (J.L.); qiaoxinsdu@126.com (X.Q.); wcy2000_zm@163.com (C.W.); 2 Jingnan Medical Area, Chinese PLA General Hospital, Beijing 100071, China; 15116953138@163.com; 3 School of Mechanical Engineering, Beijing Institute of Technology, Beijing 100081, China

**Keywords:** brain signals, brain-controlled vehicles, command decoding algorithm, driver–vehicle interfaces

## Abstract

Directly applying brain signals to operate a mobile manned platform, such as a vehicle, may help people with neuromuscular disorders regain their driving ability. In this paper, we developed a novel electroencephalogram (EEG) signal-based driver–vehicle interface (DVI) for the continuous and asynchronous control of brain-controlled vehicles. The proposed DVI consists of the user interface, the command decoding algorithm, and the control model. The user interface is designed to present the control commands and induce the corresponding brain patterns. The command decoding algorithm is developed to decode the control command. The control model is built to convert the decoded commands to control signals. Offline experimental results show that the developed DVI can generate a motion control command with an accuracy of 83.59% and a detection time of about 2 s, while it has a recognition accuracy of 90.06% in idle states. A real-time brain-controlled simulated vehicle based on the DVI was developed and tested on a U-turn road. Experimental results show the feasibility of the DVI for continuously and asynchronously controlling a vehicle. This work not only advances the research on brain-controlled vehicles but also provides valuable insights into driver–vehicle interfaces, multimodal interaction, and intelligent vehicles.

## 1. Introduction

Vehicles controlled directly by brain signals rather than using limbs are called brain-controlled vehicles [1]. Brain-controlled vehicles can provide an alternative or novel way for individuals with severe neuromuscular disorders to expand the scope of their life activities and thus enhance their quality of life. Brain signals-based driver–vehicle interfaces (DVIs), which are responsible for translating brain signals into driving commands, are the core part of these brain-controlled vehicles. Compared to DVIs based on eye tracking [2,3,4] and speech recognition [5,6,7], brain signals-based DVIs do not need speech input and have low or no requirements in terms of neuromuscular control capabilities. Therefore, they are especially desirable for severely disabled people (or wounded soldiers on battlefields) to operate a vehicle. Even for healthy drivers or special service drivers, such as military vehicle drivers, brain signals-based DVIs could represent a new form of driving assistance. For example, soldiers can use such DVIs to perform a secondary task (e.g., turning on/off a device), while they need to use limbs to control a vehicle and cannot free their limbs to perform a secondary task [8,9,10]. Thus, brain signals-based DVIs have important value for military and civil applications.

Recently, due to the low cost and convenient use of electroencephalogram (EEG) recording in practice [11,12,13], EEG signals have been explored for the development of DVIs. According to the type of output command, EEG-based DVIs can be classified into two groups: (1) DVIs for selecting a task and (2) DVIs for issuing a motion control command. For the first type, users first need to use brain signals to choose a task command from a predefined list and convey it to an autonomous control system, and then the autonomous control system has the responsibility of performing this selected task.

For the former, users first need to use brain signals to select a particular task from an available list of predefined tasks, which is transmitted to an autonomous control system, and then the autonomous control system has the responsibility of executing the selected task. Gohring et al. [14] applied a commercial event-related desynchronization/event-related synchronization (ERD/ERS)-based brain–computer interface (BCI) product from the Emotiv company to build a DVI that allows for path selection at the intersection of a road. Upon making a selection, the autonomous vehicle will perform the chosen direction and reach the desired destination. Bi et al. [15] also built a DVI using P300 potentials, which enable the user to select a destination from a list of nine predefined destinations. The brain-controlled vehicle can first detect the desired destination of the user through their brain signals, and then a vehicle autonomous navigation system is employed to transport the user to the desired destination. Fan et al. [16] further enhanced the destination detection performance of the DVI that was developed in [15] by combining steady-state visual evoked potentials (SSVEP) with P300 signals. However, a major weakness of this type of brain-controlled vehicle is that once the task is selected, the autonomous control system takes over the vehicle until the selected task is completed. Therefore, the user cannot further control the vehicle during vehicle motion.

For the second type of brain-controlled vehicle, users apply EEG signals to generate specific vehicle control commands (e.g., direction and speed) to control the vehicles to reach their destinations. Compared with the first type of vehicle, the second type allows drivers to control vehicle motion throughout the whole process of driving. Gohring et al. [14] developed a DVI based on a commercial ERD/ERS BCI product assisted by vehicle intelligence to perceive environments. While the vehicle will collide with objects or travel out of the safety zone, the intelligent system imminently executes braking action according to information obtained from its sensors. The experimental results from one subject showed that it is feasible to use EEG signals with the assistance of vehicle intelligence to discontinuously control an intelligent vehicle. However, they have not shown the feasibility of using EEG signals alone to continuously control a vehicle without the assistance of vehicle intelligence. To address this, Bi et al. [1] built and combined two BCIs (i.e., a novel SSVEP BCI and a BCI based on the alpha wave of EEG signals) to develop a DVI enabling the user to issue commands, including turning left, turning right, going forward, and starting and stopping. An online driving task, which included lane-keeping and avoiding obstacles, was tested on a U-shaped road. The experimental results showed that it is feasible to use EEG signals alone to continuously control a vehicle.

However, to the best of our knowledge, no studies have explored how to develop a P300-based DVI to generate a motion control command for continuously controlling a vehicle because the traditional P300 paradigm cannot issue a command rapidly and accurately [15,17,18,19] which is necessary for real-time continuously controlling of a dynamic system. However, P300-based DVIs have some advantages over other types of EEG-based DVIs. Compared to ERD/ERS-based DVIs, P300-based DVIs can require less training, and this is important for practical applications [20]. Compared to SSVEP-based DVIs, P300-based DVIs may generate fewer false alarms when users look at the interface but do not want to issue a command, and they annoy subjects less [21,22,23]. Also, it should be noted that SSVEP stimuli (particularly low-frequency SSVEP stimuli) tend to make users more annoyed, and high-frequency SSVEP responses are weaker and harder to accurately detect [24,25]. Moreover, P300-based DVIs can be applied to users to whom the SSVEP or ERD/ERS BCIs are not applicable due to BCI illiteracy [26]. 

Although some studies explored how to use P300 potentials to control a wheelchair or humanoid robot by issuing motion control commands [27,28], the former are task-level controlled systems, and the latter move slowly and discontinuously. Furthermore, controlling a vehicle using EEG signals is more challenging than controlling a mobile robot since brain-controlled vehicles are more complicated due to their dynamic characteristics and travel at higher speeds [1]. Mezzina and Venuto et al. [29,30] proposed a P300-based BCI to control an acrylic prototype car with ultrasonic sensors. The limitation of the two studies is that (1) the prototype car is really too small to simulate a realistic driving experience; (2) the travel speed is too low (i.e., 10 cm/s); (3) the researchers did not take the asynchronous control into consideration; and (4) the researchers did not set the controlling task and did not test the online driving performance. 

We have developed a P300-based BCI that can rapidly and accurately decode human intention offline [31], which has not been applied to develop a brain-controlled vehicle. More importantly, the asynchronous control was not taken into consideration, which can reduce the workload by detecting the non-control willingness of the user in practice [32,33,34,35]. In this paper, we aim to develop a novel asynchronous P300-based DVI for continuously controlling a brain-controlled vehicle and validate the proposed DVI by using driver-in-the-loop online experiments. This work not only advances research on brain-controlled vehicles but also provides valuable insights into DVI, multimodal interactions, and intelligent vehicles. 

The main contributions of this study are as follows:

(1) This work is the first to use P300 potentials to develop a DVI to enable users to control a vehicle continuously and asynchronously.
(2) The control command decoding algorithm, including the asynchronous function, was proposed for the development of the P300-based DVI.
(3) We tested and validated the feasibility of the P300-based DVI for controlling a brain-controlled vehicle using a driver-in-the-loop online experiment.

## 2. Materials and Methods

### 2.1. System Architecture

**Figure 1:**
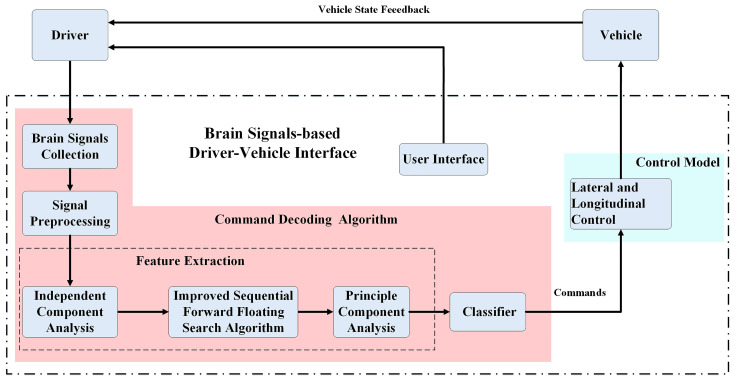
Structure of brain signals-based driver-vehicle interface.

As shown in Figure 1, the proposed brain signals-based DVI includes three main components: (1) user interface, (2) command decoding algorithm, and (3) control model. The DVI can provide four types of control, including turning left, turning right, switching speed, and going forward. The working procedure is as follows. According to the vehicle state and surroundings information, brain-controlled drivers decide whether to send control commands or not to ensure the vehicle travels safely. If the direction or speed of the vehicle needs to be changed, the driver needs to pay attention to the corresponding command characters in the user interface. Otherwise, they do not need to pay attention to any of the characters. Meanwhile, the detection system constantly collects EEG signals. The collected EEG signals are first preprocessed and then transformed into independent components (ICs) using independent component analysis (ICA). After that, the improved sequential forward floating search (ISFFS) algorithm is used to select the optimal ICs. These selected ICs are employed as original features. Then, the features compressed from the original features using principal component analysis (PCA) are fed into a classifier. Finally, the output of the classifier is transformed into the inputs of the control model.

### 2.2. User Interface

**Figure 2:**
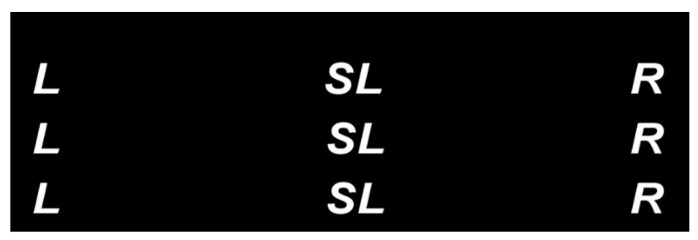
User interface, where L, R, and S (SL or SH) represent turning left and right and switching speed, respectively.

As shown in Figure 2, we designed a novel user interface in our previous [31], where L, R, and S (including SL and SH for switching to low and high speeds, respectively) represent turning left and right and switching speed commands. In the interface, nine letters are distributed in a 3 × 3 matrix, and every three of the same letters denote one type of command. These nine letters flash sequentially in a random order every round, similar to the odd-ball P300 paradigm [36,37]. Each round refers to a flashing cycle of the nine characters, in which each letter flashes and lasts for 120 ms only once. The interval between two consecutive letter flashings is set to zero in the paradigm. Thus, the time period for one complete round is 1.08 s (120 ms × 9).

Intuitively, the total flashing repetition number of every command (i.e., L, S, or R) is three in one round and six in two rounds because every three of the same letters denote one type of command. However, in some random flashing sequences, two or all of the three same characters corresponding to one command in a round may successively flash in a single round. On the basis of the findings in [38,39,40], in case the stimulus onset asynchrony (SOA) is less than 500 ms, an attention blink occurs. Therefore, for a command in these flashing sequences, only the first corresponding flashing character may catch the user’s attention, and the last one or two may not. Therefore, compared to traditional paradigms, the segmentation and summation rule of the continuous EEG data needs to be modified to avoid extracting useless information (for more detail, see Section 2.3.1).

### 2.3. Command Decoding Algorithm

**Figure 3:**
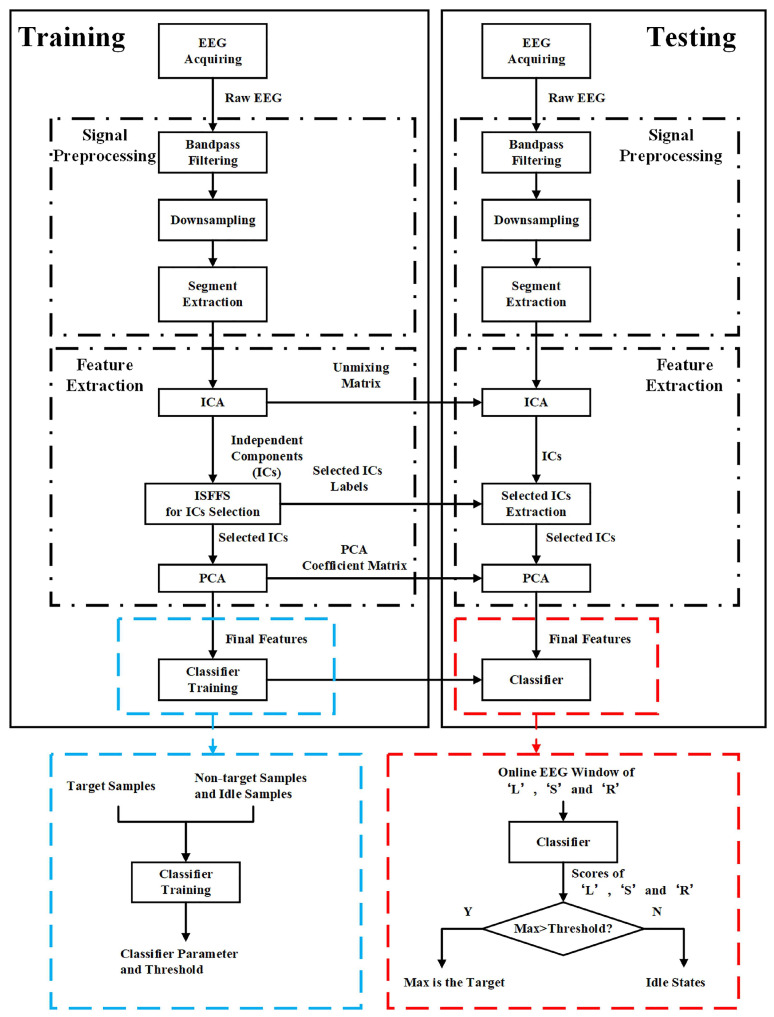
Signal flowchart of the command decoding algorithm.

Figure 3 shows the signal flow chart of the command decoding algorithm. The flow chart includes both the training and testing phases. Each phase includes signal preprocessing, feature extraction, and classification. In the training phase, after the EEG signals were collected, the unmixing matrix of ICA, labels of optimal ICs selected by using ISFFS, transformation matrix of PCA, parameters of the classifier, and threshold were determined for online command decoding. In the testing phase, all of the trained parameters are utilized to decode the desired command from EEG signals for real-time control of a vehicle.

#### 2.3.1. Training Phase

**Figure 4:**
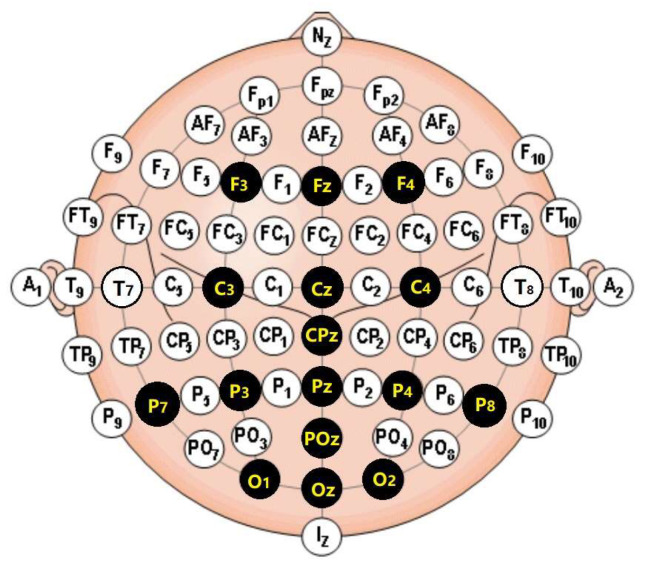
Channels used to collect EEG signals were marked in black disks with yellow text.

Data collection: Sixteen standard locations are set according to the international standard 10–20 montage. EEG signals are collected from each of these locations through a 16-channel amplifier made by SYMTOP, as shown in Figure 4. The mean potential of the left and right earlobes is set as the reference potential to other channels. EEG signals are amplified and digitalized with a sampling rate of 1000 Hz, and line noise is removed using a power-line notch filter.

**Figure 5:**
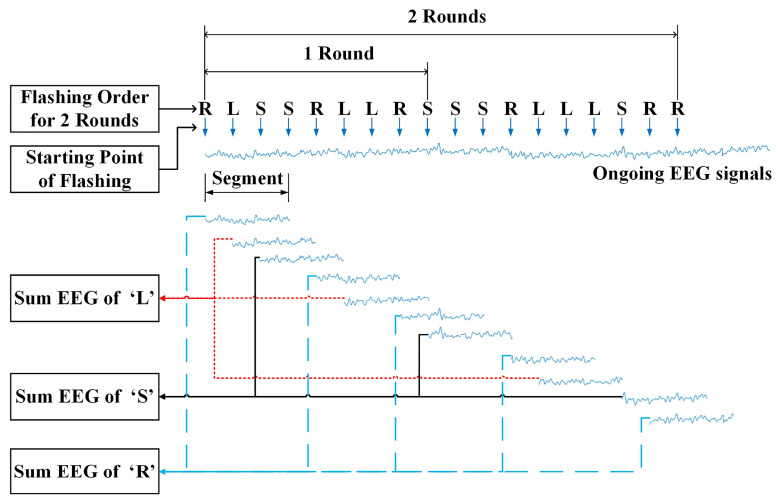
Illustration of the EEG segment extraction procedure.

Preprocessing: Once the interface completes all rounds of flashing, the collected EEG data are filtered from 0.53 Hz to 15 Hz using a bandpass filter and down-sampled by a factor of two. EEG segments are then extracted for each command according to the following two rules. Rule 1: Command labels of all characters in all rounds are recorded, and all the corresponding EEG signals of all letters are segmented from the onset of each letter flashing to post-stimulus T. Rule 2: If there exist two, three or more successive same flashing letters in each command label, only the first letter is considered as an effective flashing letter and the corresponding EEG segment is extracted for further processing; otherwise all the flashing letters are considered as effective and all of the EEG segments are used for decoding commands. Figure 5 shows the illustration of the segment extraction procedure. Finally, the extracted EEG segments associated with each command are summed.

ICA: Original features are extracted from the IC domain. The extracted 16-channel EEG segment associated with each command is taken as the input of ICA, and ICs are then obtained by

(1) Y(t)=UX(t)

where $X(t)={\left[X_{1}(t),X_{2}(t),\ldots ,X_{i}(t),\ldots ,X_{L}(t)\right]}^{T}$ represents the extracted EEG segment with a length of 512 ms, as shown in Figure 5 from the *i*th channel, and *L* represents the number of all channels and is set as 16 in this work. $Y(t)={\left[Y_{1}(t),Y_{2}(t),\ldots ,Y_{i}(t),\ldots ,Y_{L}(t)\right]}^{T}$ represents the independent component vector, *t* denotes the sampling time instance, and $U\in {ℜ}^{L\times L}$ represents the unmixing matrix, which can be obtained by using the Infomax method [41].

ISFFS: ISFFS is an improved SFFS and is used to select the optimal ICs from all ICs proposed in our previous study [31]. The feature pool *F* can be written as

(2) $$\begin{array}{l} F=\left\{f_{1},f_{2},\ldots ,f_{k},\ldots ,f_{L}\right\}\\ f_{k}=[a_{k}(1),a_{k}(2),\ldots ,a_{k}(i),\ldots ,a_{k}(N)] \end{array}$$

where $f_{k}$ represents the feature vector associated with *k*th IC; $a_{k}(i)$ represents the *i*th time domain feature of $f_{k}$; *L* represents all the number of ICs and is equal to the number of all channels; *N* represents the dimensionality of every IC and is set to 256 due to the down sample factor of 2.

The SFFS is a bottom-up search procedure. During the optimal feature subset search procedure, the most significant feature is included in the current optimal subset. Meanwhile, the least significant feature of the current optimal subset will be excluded. Considering the long period of high computational complexity, especially for time domain features with a high resampling rate of EEG signals in this study, we apply principal component analysis to decrease the dimensionality of the time domain features and thus significantly reduce the computational time and make it feasible in practice. This method is subsequently referred to as ISFFS. More details about the ISFFS algorithm can be seen in our previous study [29].

Classifier: After the optimal ICs are obtained, the PCA is used to decrease the dimensionality of the selected features, which are obtained by concatenating the optimal ICs. Assume that the number of optimal ICs selected by ISFFS for each subject is *K,* and thus, the number of the original features is *K* × *N*. The components with the highest *P* eigenvalues are chosen as feature weights, and new features can be presented as $x={\left[x(1),\;x(2),\;\ldots ,\;x(i),\;\ldots ,x(P)\right]}^{T}$, which can cover more than 95% information of all the original features. *P* is set to 50 for all users in this work to meet the above requirement. 

We apply both Fisher linear discriminant analysis (FLDA) and the support vector machine (SVM) to build the classifiers. The classifier built by the FLDA can be denoted as

(3) $$y=w^{T}x$$

where *w* represents the projection direction. The value of threshold η is determined by the receiver of curve (ROC) method. If the score *y* is larger than η, the sample is classified into the target class; otherwise, the sample will be classified into the non-target class or idle class.

The classifier built using the SVM with the radial basis function (RBF) as the kernel function can be represented as

(4) $$y=\sum_{i=1}^{n}w_{i}\exp\left(-g{\left\|x_{i}-x\right\|}^{2}\right)+b$$

where $x_{i}$ is the *i*th support vector (SV) of the classifier, $w_{i}$ is the weight of the *i*th SV of the classifier, *n* is the number of the SV of the classifier, *g* is the width of the RBF of the classifier, and *b* is the bias of the classifier. We used the LIBSVM software (Version 2.0) library proposed by Chang and Lin [42] to train the parameters of the SVM classifier.

#### 2.3.2. Testing Phase

As shown in Figure 3, the testing experiment is performed using the following three steps. First, EEG signals associated with three types of command characters are extracted, and the corresponding features are computed in each cycle. Next, these features are fed into the classifier, and scores associated with three types of commands are all obtained. Third, if the maximum of the three scores is larger than η, then the corresponding type of command character is recognized as the target. If the maximum is smaller than η, then an idle state (i.e., a going forward command) is issued.

### 2.4. Control Model

Two control models, lateral and longitudinal, were designed in the proposed DVI. The lateral control model is defined as follows:

(5) $$\begin{array}{l} \theta (n)=\min\{\left|\theta (n-1)+\Delta \theta \times \Omega (n)\right|,\theta_{\max}\}\\ \Omega (n)\in \{-1,0,1\}\\ \Delta \theta =\alpha \\ \theta (0)=0^{\circ }\\ \theta_{\max}=90^{\circ }\\ n\geq 1 \end{array}$$

where θ(n) represents the steering angle at the *n*th update and Ω(n) represent the output command of the decoding algorithm, with Ω(n)=−1 for turning right, Ω(n)=0 for going forward, and Ω(n)=1 for turning left. In addition, Δθ is a positive constant angle value set as α. The value of α can be calibrated for different subjects (e.g., 15°, 20°, or 30°) before the formal online experiment.

The longitudinal control model is defined as follows:

(6) $$\begin{array}{l} V(n)=\left\{\begin{array}{l} V(n-1),\;\mathrm{if}\;\Omega (n)\neq 2\\ V_{1},\;\mathrm{if}\;\Omega (n)=2,\;V(n-1)=V_{2}\\ V_{2},\;\mathrm{if}\;\Omega (n)=2,\;V(n-1)=V_{1} \end{array}\right.\\ V(0)=V_{1}\\ n\geq 1 \end{array}$$

where V(n) represents the speed. $V_{1}$ is set to be about 7 km/h and $V_{2}$ is set to be about 8 km/h. Ω(n) represents the output command with Ω(n)=2 for the switching speed.

## 3. Experiment

### 3.1. Subjects

Six healthy subjects (aged between 21 and 25) participated in the experiment. All of the subjects had no history of brain disease and had normal or corrected-to-normal vision. The study adhered to the principles of the 2013 Declaration of Helsinki and was conducted in accordance with the Declaration of Helsinki. All subjects signed an informed consent form after the experiment’s purpose, required tasks, and possible consequences of participation were explained.

### 3.2. Experimental Platform

**Figure 6:**
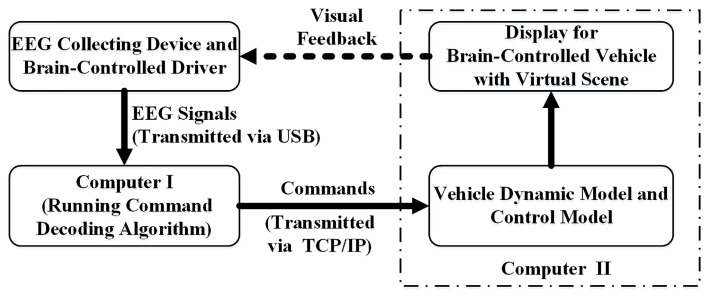
Experimental platform.

As shown in Figure 6, the experimental platform includes a simulated vehicle with a virtual driving scene and the proposed DVI. The simulated vehicle was supported by 14-DOF vehicle dynamics from CarSim software (version 8.02), and the virtual scene was developed with Matlab/Simulink. The command decoding algorithm is composed of an EEG amplifier for collecting EEG signals and signal processing and a classification algorithm coded in C. The communication system between the computer running the command decoding algorithm based on EEG signals and the computer running the control model and virtual vehicle was built with the Transmission Control Protocol/Internet Protocol (TCP/IP).

### 3.3. Experimental Procedure

Prior to conducting the experiments, some preparations were made. We adjusted the distance between the subjects and the screen displaying the user interface to make subjects feel comfortable. All of the subjects were informed of the entire experimental procedure to make them fully aware of the experiment protocol. Electrodes were appropriately attached at corresponding locations, and all the contact impedances between the scalp and electrodes were adjusted to be below 10 kΩ. In the online experiment, the user interface was presented as a head-up display system. This allowed the users to pay attention to the interface to issue control commands as well as observe the road conditions in case of traveling out of the road. All apparatuses were examined before EEG collection to ensure normal operation during the experiment. 

The experiment consisted of two parts: (1) training and testing the command decoding algorithm and (2) testing the developed brain-controlled vehicle based on the proposed brain signals-based DVI. In the training part, the training data were collected and used to determine the parameters and test the performance of the command decoding algorithm. EEG data from all three rounds in one trial were collected for evaluation of the performance of the command decoding algorithm offline, given different round numbers. 

**Figure 7:**
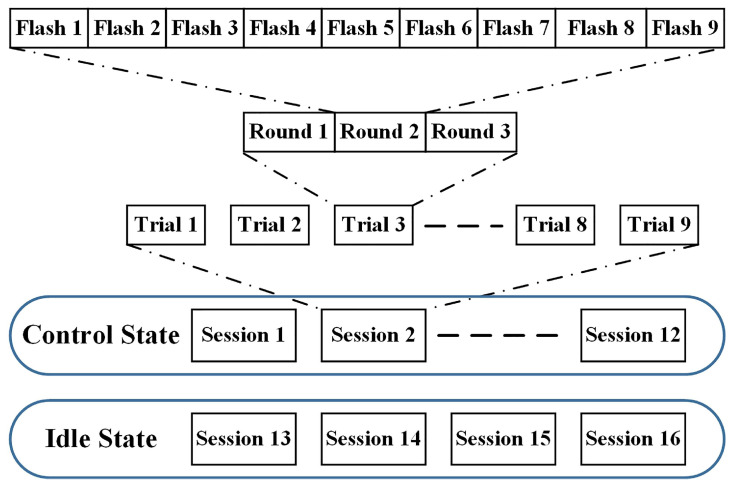
Data collection for control and idle states.

As shown in Figure 7, the data collection consisted of two phases: the first phase for control states (target and nontarget samples) and the second phase for idle states. After twelve sessions were completed for control command data collection, four sessions were conducted for idle state data collection. Each session included nine trials, and every trial included three rounds. In the first phase, the subjects concentrated their visual attention on the user interface and counted the flashing number of the predefined target letter immediately every time it flashed during the trials until the session was concluded. In the second phase, subjects were asked not to attend to any characters in every session while the user interface worked all the time. A few minutes were permitted for the subjects to rest between two consecutive sessions for each phase. 

In total, we obtained 108 target samples, 216 non-target samples, and 108 idle samples. These samples were classified into two classes. Class 1 consisted of all 108 target samples. Class 2 consisted of all 216 non-target samples when we evaluated the algorithm without considering the idle states. When we took idle states into consideration, Class 1 consisted of all 108 target samples, and Class 2 consisted of all 216 non-target and 108 idle samples. The whole offline training phase was completed within 40 min. 

During the part of testing the brain signals-based DVI, subjects were required to perform the driving task: controlling the simulated vehicle to travel along the centerline of the road from the starting point to the destination using the proposed DVI. Subjects should try their best to drive the vehicle to stay inside the road boundaries while driving. If the vehicle travels out of the road boundaries, subjects should try their best to control the vehicle back to the road. Before the formal testing, subjects practiced some runs to learn how to control the brain-controlled vehicle by using the brain signals-based control system. Every subject performed five runs and was given a few minutes to rest between two consecutive runs.

## 4. Results and Discussion

### 4.1. EEG Response under the Paradigm

**Figure 8:**
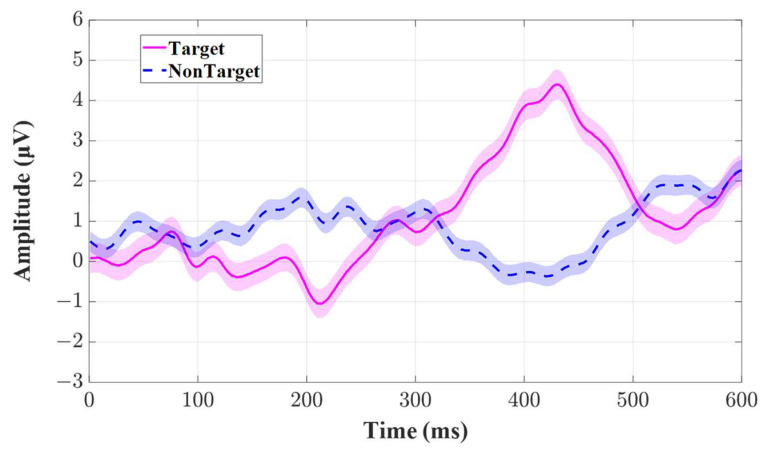
The ground-average EEG responses across all the participants to the target (red solid line) and non-target stimuli (blue dash line) at channel Cz. The shadow areas represent the standard errors at the corresponding sampling time point from the onset of the flashing to the post-stimuli 600 ms.

Figure 8 shows the ground-average EEG responses across all the participants to the target and non-target stimuli at channel Cz. The horizontal axis represents the time from the onset of the flashing to the post-stimuli 600 ms, and the vertical axis represents the amplitude of the EEG signals. The red solid line denotes the EEG wave morphology elicited by the target stimuli, and the blue dashed line denotes the EEG wave morphology elicited by the non-target stimuli. The shadow areas around the lines represent the standard errors corresponding to the sampling time point. We can see that the amplitude of EEG wave morphology ranged from −2 μV to 5 μV. The typical P300 component is elicited with an evident positive going wave at about 420 ms, and the N200 component is also elicited near 200 ms. Both components are present in the EEG response to the target stimuli, yet they are absent in the EEG response to nontarget stimuli.

### 4.2. Performance of the Proposed DVI

We ran 100 × 6-fold cross-validations to evaluate the performance of the proposed BCI based on the samples as mentioned in section III (C) and took the idle states into consideration. Thus, the used samples for training and testing included 108 target samples, 216 non-target samples, and 108 idle samples. 

Table 1 and Table 2 show the accuracy of the proposed DVI with FLDA and SVM classifiers as a function of the detection time. We can see that for the two classifiers, the accuracies in control and idle states both increase over the detection time. The FLDA significantly outperforms the SVM in both control states and idle states on average, given the different detection times (*p* = 0.005, *p* = 0.024, *p* = 0.043, *p* = 0.039, *p* = 0.025, *p* = 0.003). 

For the DVI with the FLDA classifier, the average accuracies with standard errors in control states are 77.3 ± 2.0%, 83.6 ± 2.6%, and 87.5 ± 2.6%, respectively, whereas the average accuracies with standard errors in idle states are 86.8 ± 1.4%, 90.1 ± 2.7%, and 92.7 ± 2.4%, respectively, given detection times of 1.08 s, 2.16 s, and 3.24 s. Furthermore, half of all subjects performed well with an accuracy near 90% in control states and higher than 93% in idle states, given a detection time of 2.16 s. In this paper, we took a detection time of 2.16 s for online control of a vehicle.

To make sure that the proposed method could be implemented in real-time without incurring a substantial delay, we calculated the computational time of the entire command decoding algorithm with an Intel^®^ Core ^TM^ i7-3770 CPU (3.40 GHz). The average computational time was about 340 ms.

### 4.3. Performance of Brain-Controlled Vehicles Based on the Proposed Novel Asynchronous Brain Signals-Based DVI

The real-time brain-controlled simulated vehicle based on the proposed DVI was developed and tested on a U-turn road for six subjects. The task completion time and mean lateral error were selected as evaluation indexes to rate the performance of the vehicles. The task completion time was defined as the time taken for the vehicle to travel from the starting point to the destination. When the task completion time was longer than twice that of the nominal time, the task was stopped and defined as a failed task. The nominal time was calculated as the length of the centerline of the road from the starting point to the destination over the maximum speed. The mean lateral error was defined as the mean of the lateral error between the trajectory of the simulated vehicle and the centerline of the road from the starting point to the destination. It should be noted that the two measures are applicable only when an experimental run is successfully completed. 

**Figure 9:**
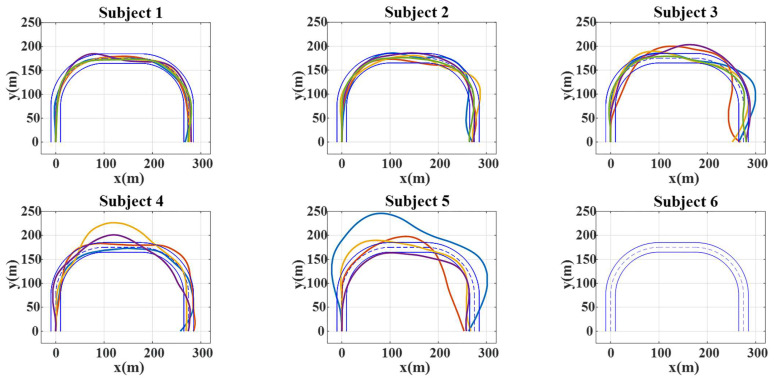
The trajectories of the developed brain-controlled vehicle based on the proposed DVI in successful runs of the consecutive 5 runs for every subject. Each thick line represents each successful run.

Figure 9 shows the trajectories of the brain-controlled vehicles based on the proposed DVI in the five consecutive runs for each subject. The two solid blue lines represent the road boundaries. Other color lines represent the vehicle trajectories of the successful tasks. Subjects 1, 2, and 3 achieved good performance in brain-controlled tasks, and they completed all five runs. Subjects 4 and 5 did not perform as well as Subjects 1, 2, and 3 and completed only four out of five runs. Subject 6 exhibited the worst performance and did not complete any of the runs. The task completion times of the subjects were 250.01 ± 2.79 s, 253.21 ± 1.68 s, 263.20 ± 3.17 s, 261.97 ± 5.64 s, and 265.33 ± 19.98 s and the mean lateral errors were 2.43 ± 0.48 m, 4.21 ± 0.92 m, 8.85 ± 2.05 s, 9.09 ± 1.91 s, and 18.2 ± 5.37 s, respectively. Subject 1 performed the task best of all subjects with the lowest mean lateral error and the lowest mean task completion time of about 250 s, which is close to the ideal task completion time of 245 s. Other subjects, especially Subjects 5 and 6, performed poorly.

The major reason for the performance difference in controlling a vehicle among the subjects is that the control command recognition accuracies in control and idle states are different between the subjects. As shown in Table 1, the accuracies in control states for Subjects 1, 2, and 3 were both near 90%, while the accuracies in control states for Subjects 5 and 6 were under 80%. The accuracies in idle states for Subjects 1, 2, and 3 were both higher than 93%, while the accuracies in idle states for Subjects 5 and 6 were under 90%. A possible reason for this disparity may have involved difficulties associated with eliciting specific brain activity patterns. It has been demonstrated that BCIs do not work for some users, which is referred to as “BCI illiteracy” [43,44,45]. 

Another possible reason for the task performance difference is that subjects need to learn how to use the proposed DVI rather than their limbs to drive the brain-controlled vehicle, which is a completely new operation mode for them. Some subjects may not learn well or need more practice. For example, although Subject 3 had the same accuracy in control states as Subject 2 and even higher performance in idle states than Subject 2 at the same detection time, the latter performed the tasks in both less time and with a lower mean lateral error in brain-controlled tracking than the former in all five runs. 

Compared to the brain-controlled vehicle reported in [1], which enabled users to issue lateral control and starting/stopping commands to control a vehicle continuously, the proposed brain-controlled vehicle can provide lateral control and switching speed commands and shows comparable performance. It should be noted that experimental results in this paper and [1] are from different subjects (four subjects used in [1] and six subjects used in this paper are not any of the same subjects). Furthermore, the proposed brain-controlled vehicle does not require subjects to issue commands by closing their eyes and thus has lower requirements for the neuromuscular control capabilities of subjects. Finally, the proposed brain-controlled vehicle may provide an alternative way for some subjects who have a relatively low accuracy in using the brain-controlled vehicle developed in [1]. For other brain-controlled systems, such as telepresence controlling an NAO humanoid robot in [28], the robot moved slowly and discontinuously and was not controlled in the first perspective. The comparison between this work and the related articles is also shown below. Table 3 shows the thorough comparison of the proposed system between this work and the related articles, including the controlled objective, whether it is a dynamic system or not, whether it is continuously controlled or not, with or without asynchronous function, active or passive BCI, and some notes, if any. Table 4 shows the thorough comparison of the performance between this work and the related articles, including the type of EEG signals, offline classification accuracy, detection time (s), online performance, and some notes, if any. Venuto et al. [29] and Mezzina et al. [30] proposed a continuously controlled dynamic system without the asynchronous function using P300 signals. The detection time is relatively low, and the online accuracy is comparable. However, the speed is relatively low, and the online driving performance is not available. 

There are some limitations in this study. First, it is necessary to expand the subject pool with more diverse subjects for data collection. Experiments with a larger and more diverse group of participants could provide a more comprehensive understanding of the system’s effectiveness and potential limitations. Second, although a higher information transfer rate (ITR) does not mean better controlling performance, there is a need to conduct a comparative analysis of ITR in real-time tasks when conducting the online experiment against other relevant studies to achieve a more comprehensive evaluation of the performance of these brain-controlled systems, like that in [30].

## 5. Conclusions

This paper developed a novel asynchronous brain signals-based DVI that can be used to control the speed and direction of a vehicle. The proposed DVI consists of a user interface, command decoding algorithm, and control model. The real-time brain-controlled simulated vehicle based on the proposed DVI was developed and tested on a U-turn road. The experimental results demonstrate the feasibility of the proposed DVI in controlling a vehicle, at least for some users who have high recognition accuracy in control command and idle states. The study is important for the future development of brain-controlled dynamic systems (especially based on event-related potentials), driver–vehicle interfaces, intelligent vehicles, and multimodal interactions with several implications. First, it highlights the difference between evaluating BCIs and brain-controlled dynamic systems. Controlling a dynamic system is more difficult and challenging. Second, compared to brain-controlled wheelchairs and robots (e.g., NAO robots), brain-controlled vehicles are more complicated, and they travel at higher speeds. Thus, the proposed method should be useful for improving the control of brain-controlled wheelchairs and robots. Third, the proposed DVI can be applied to perform a secondary driving task (e.g., turning on/off a GPS device) while healthy drivers use limbs to control a vehicle, which provides new insights into multimodal interaction and intelligent vehicles. 

Our future work aims to improve the recognition accuracy of the DVI by using deep learning methods [49,50], validate the proposed DVI with more diverse subjects in various driving scenes, design an assistive controller to improve the overall driving performance and evaluate the impact of fatigue on DVI system performance, ideally by comparing it to real driving tasks during fatigue states. We will consider user satisfaction and subjective workload assessment to offer a more holistic evaluation. We also plan to apply the proposed method to control other dynamic systems and extend the proposed DVI to perform a secondary driving task while healthy drivers use limbs to control a vehicle.

## Figures and Tables

**Table 1:** The accuracy in the control state and idle state for the FLDA as a function of detection time across all subjects (%).

Subject	Control State			Idle State		
	1.08 s	2.16 s	3.24 s	1.08 s	2.16 s	3.24 s
1	84.42	90.71	95.44	91.44	96.76	99.84
2	83.15	89.19	92.15	86.78	93.64	98.23
3	77.32	88.97	92.43	91.06	97.01	96.03
4	73.20	81.42	84.72	81.38	88.44	90.60
5	72.96	77.31	82.72	84.81	78.69	83.98
6	73.00	73.96	77.72	85.29	85.82	87.48
Mean ± std. error	77.34 ± 1.96	83.59 ± 2.62	87.53 ± 2.55	86.79 ± 1.45	90.06 ± 2.67	92.69 ± 2.36

**Table 2:** The accuracy in the control state and idle state for the SVM as a function of detection time across all subjects (%).

Subject	Control State			Idle State		
	1.08 s	2.16 s	3.24 s	1.08 s	2.16 s	3.24 s
1	80.54	87.89	94.44	87.46	91.36	95.44
2	73.70	88.49	94.02	85.38	86.45	90.76
3	64.14	74.03	83.67	80.59	85.40	88.57
4	63.96	76.29	79.47	81.62	82.36	83.75
5	58.73	68.25	72.70	80.58	81.10	82.97
6	50.85	59.03	69.90	75.35	77.60	79.80
Mean ± std. error	65.32 ± 3.94	75.66 ± 4.25	82.37 ± 3.87	81.83 ± 1.58	84.05 ± 1.78	86.88 ± 2.15

**Table 3:** A thorough comparison of the proposed system between this work and the related articles.

Literature	Controlled Objective	Dynamic System	Continuously Control	Asynchronous	Active or Passive	Notes
Fan et al. [16]	Simulated Vehicle	No	No	No	Active	-
Venuto et al. [29]	Remotely Driving Mechanical Devices	Yes	Yes	No	Active	With ultrasonic sensors
Mezzina et al. [30]	An acrylic prototype car	Yes	Yes	No	Active	With peripheral sensors
Teng et al. [46]	Simulated Vehicle	Yes	Yes	Yes	Passive	-
Bi et al. [47]	Simulated Vehicle	No	No	No	Active	-
Bai et al. [48]	Speller	No	No	No	Active	-
Our current work	Simulated Vehicle	Yes	Yes	Yes	Active	-

**Table 4:** A thorough comparison of the performance between this work and the related articles.

Literature	Type of EEG Signals	Offline Classification Accuracy	Detection Time (s)	Online Performance	Notes
Fan et al. [16]	P300	-	26	99%	-
Venuto et al. [29]	P300	-	1.03	69.54% for direction selection	Online driving performance was not available.
Mezzina et al. [30]	P300	-	2.8	84.28 ± 0.87%	Speed at 10 cm/s; Online driving performance was not available.
Teng et al. [46]	Emergency-related EEG signals	94%	0.42	-	-
Bi et al. [47]	P300	84.72%	2.16	-	-
Bai et al. [48]	P300 and SSVEP	96.86%	39.3	94.29%	Speller
Our current work	P300	83.59 ± 2.62% for control state; 90.06 ± 2.67% for idle state	2.16	Details were shown in Section 4.3	-

## Data Availability

All data included in this study are available upon reasonable request by contacting the corresponding author.
